# Supplementary material for: Biosensor Guided Polyketide Synthases Engineering for Optimization of Domain Exchange Boundaries
Source: Nat Commun. 2023 Aug 12;14:4871. doi: 10.1038/s41467-023-40464-x (PMC10423236; doi:10.1038/s41467-023-40464-x)
Supplement: Supplementary file 1 — Supplementary Information [file 41467_2023_40464_MOESM1_ESM.pdf]

Biosensor Guided Polyketide Synthases Engineering for Optimization of Domain Exchange Boundaries

Supplementary Information

Englund *et al.*

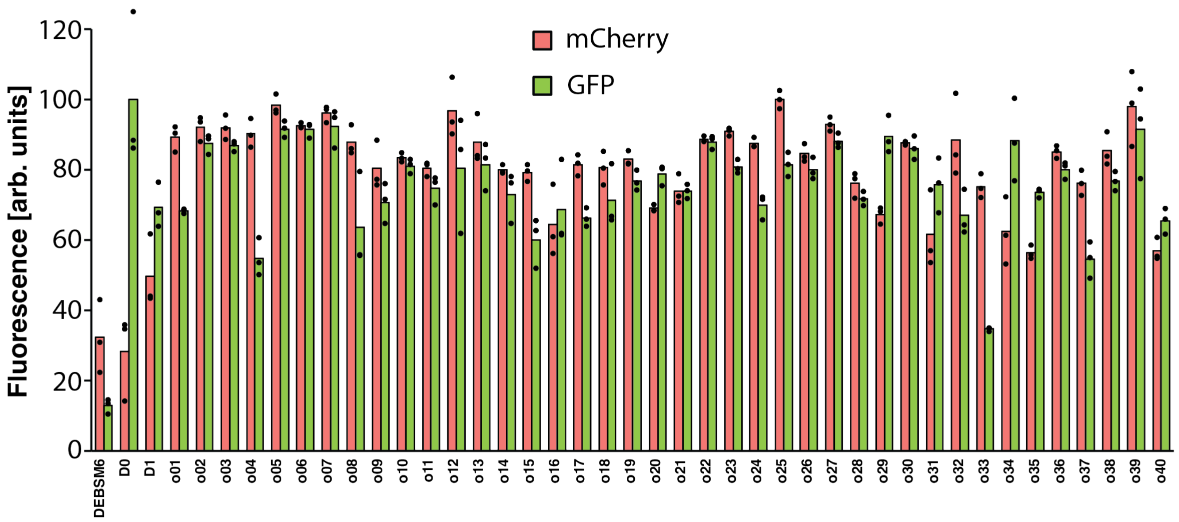

Supplementary Fig. 1. Biosensor measurement of 40 high solubility colonies. The figure shows the same data as presented in Fig. 4a but mCherry and GFP are displayed separately. All variants were expressed Δ*arsB*::P_{ibp} GFP and induced with 250 µM IPTG. Data is presented as mean values of three biological replicates, dots are individual data points. Source data is available in Source Data file. Arb. units = arbitrary units.

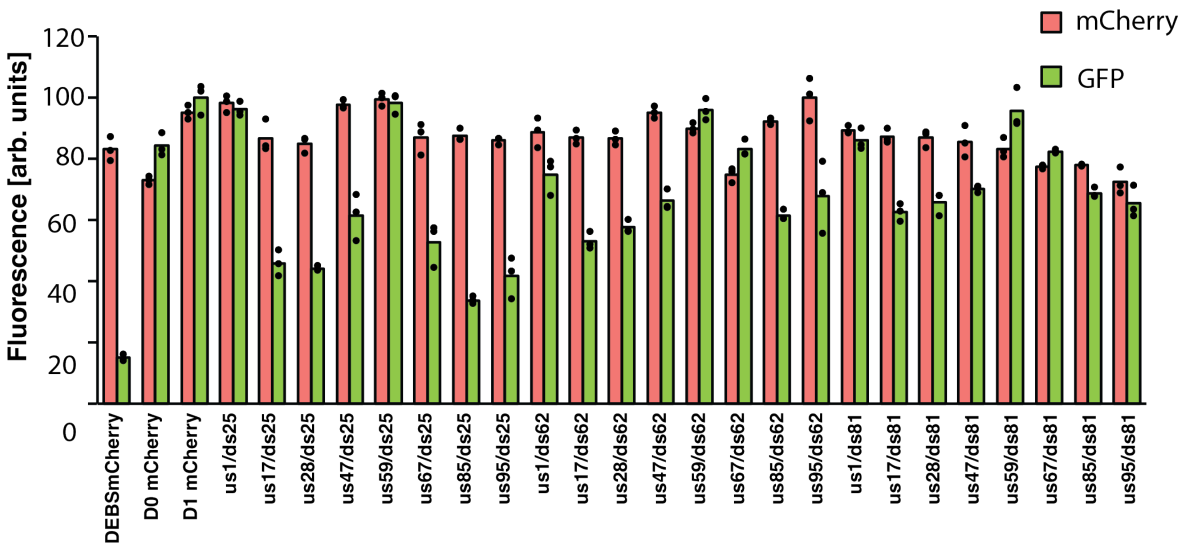

Supplementary Fig. 2. Testing amount of synergy between KS-AT and post-AT linker junctions on solubility. The figure displays the same data as presented in Fig. 5a but mCherry and GFP are displayed separately. All variants were expressed Δ*arsB*::P_{ibp} GFP and induced with 250 µM IPTG. Data is presented as mean values of three biological replicates, dots are individual data points. Source data is available in Source Data file. Arb. units = arbitrary units.

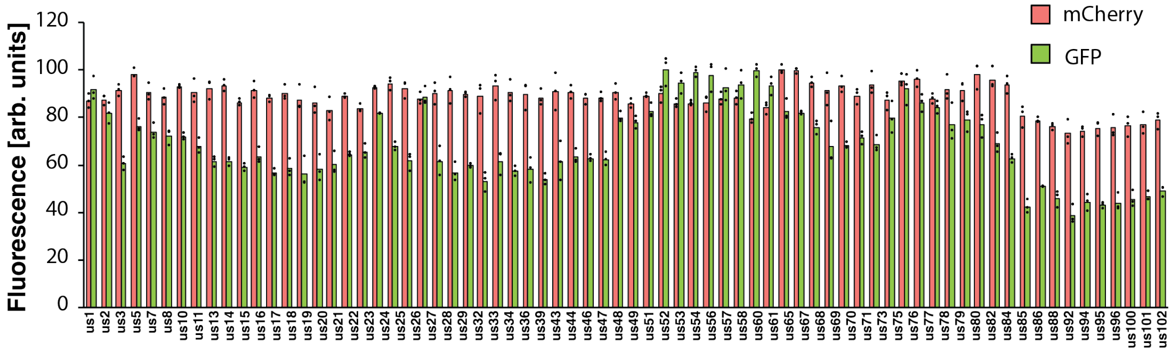

Supplementary Fig. 3. Measuring effect of EpoAT domain exchange into DEBSM6 in KS-AT linker junction positions. The data is the same as presented in Fig. 5b for EpoAT but mCherry and GFP are displayed separately. All versions were expressed Δ*arsB*::P_{ibp} GFP and induced with 250 µM IPTG. Data is presented as mean values of three biological replicates, dots are individual data points. Source data is available in Source Data file. Arb. units = arbitrary units.

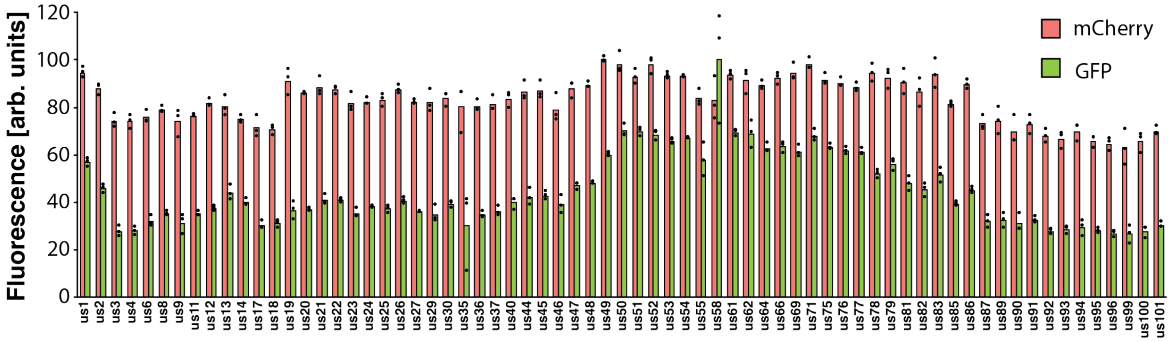

Supplementary Fig. 4. Measuring effect of TiaAT domain exchange into DEBSM6 in KS-AT linker junction positions. The data is the same as presented in Fig. 5b for TiaAT but mCherry and GFP are displayed separately. All versions were expressed Δ*arsB*::P_{ibp} GFP and induced with 250 µM IPTG. Data is presented as mean values of three biological replicates, dots are individual data points. Source data is available in Source Data file. Arb. units = arbitrary units.

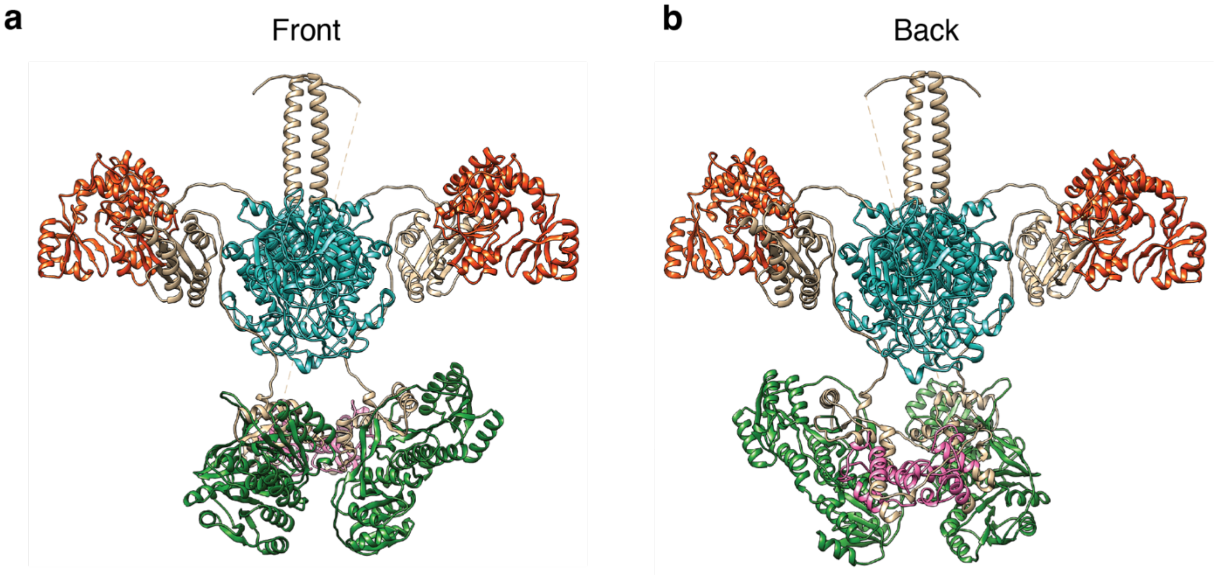

Supplementary Fig. 5: AlphaFold structure model of DEBSM6 without TE domain with domains color coded. Domain boundaries are predicted using PKS/NRPS prediction software (http://nrps.igs.umaryland.edu/)[1] and color coded as follows: KS = blue, AT = orange, KR + ψKR = green, ACP = pink, linker sequence = beige. (a) Front and (b) back view are presented to show position of ACP behind KR.

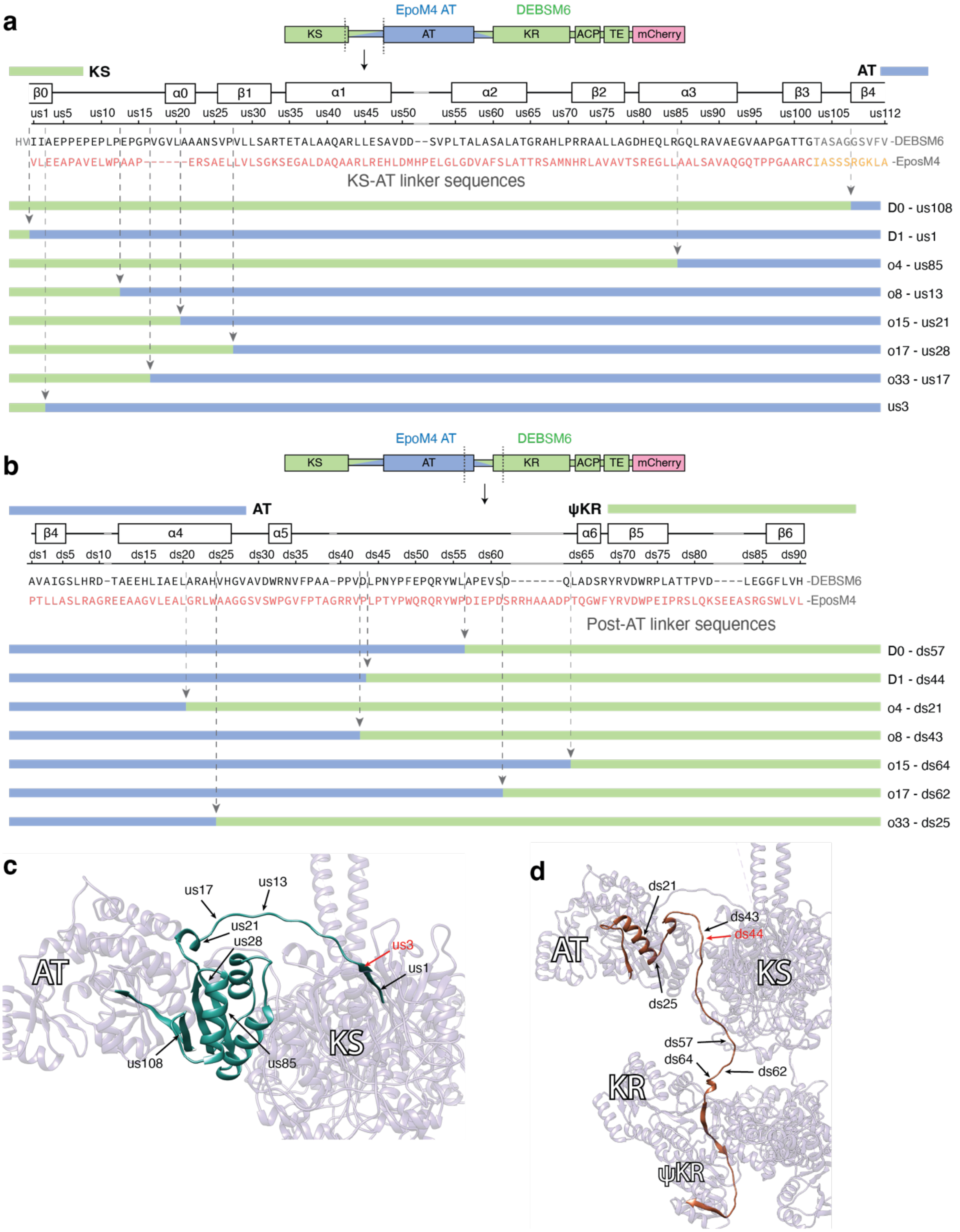

Supplementary Fig. 6: The domain junction positions displayed on sequence alignments and structural models of selected variants. (a) Junction positions in the KS-AT linker and (b) post-AT linker. DEBSM6 sequence in black, EpoM4 in red and sequences outside the selected linker region are gray and yellow. (c, d) Positions of junctions on DEBSM6 AlphaFold model. Junctions marked in red are our updated recommendation for domain exchange junctions.

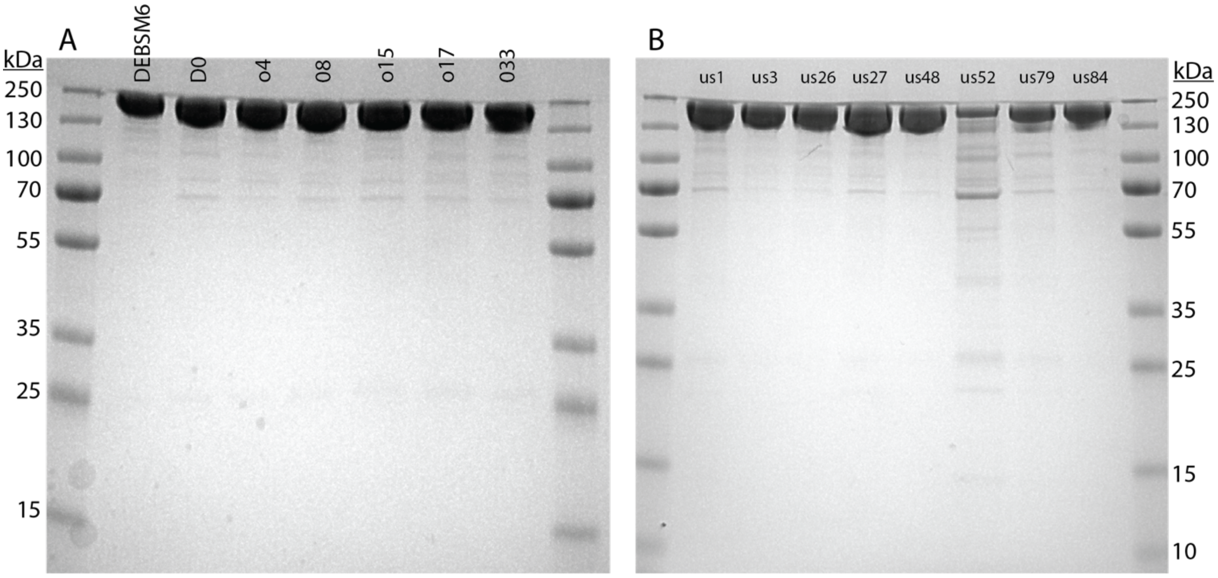

Supplementary Fig. 7. SDS-PAGE of purified proteins used in *in vitro* measurements from Fig. 4d, e in panel (a) and Fig. 5e, f in panel (b). 5 µg of protein was loaded in each well. The experiment was run only once.

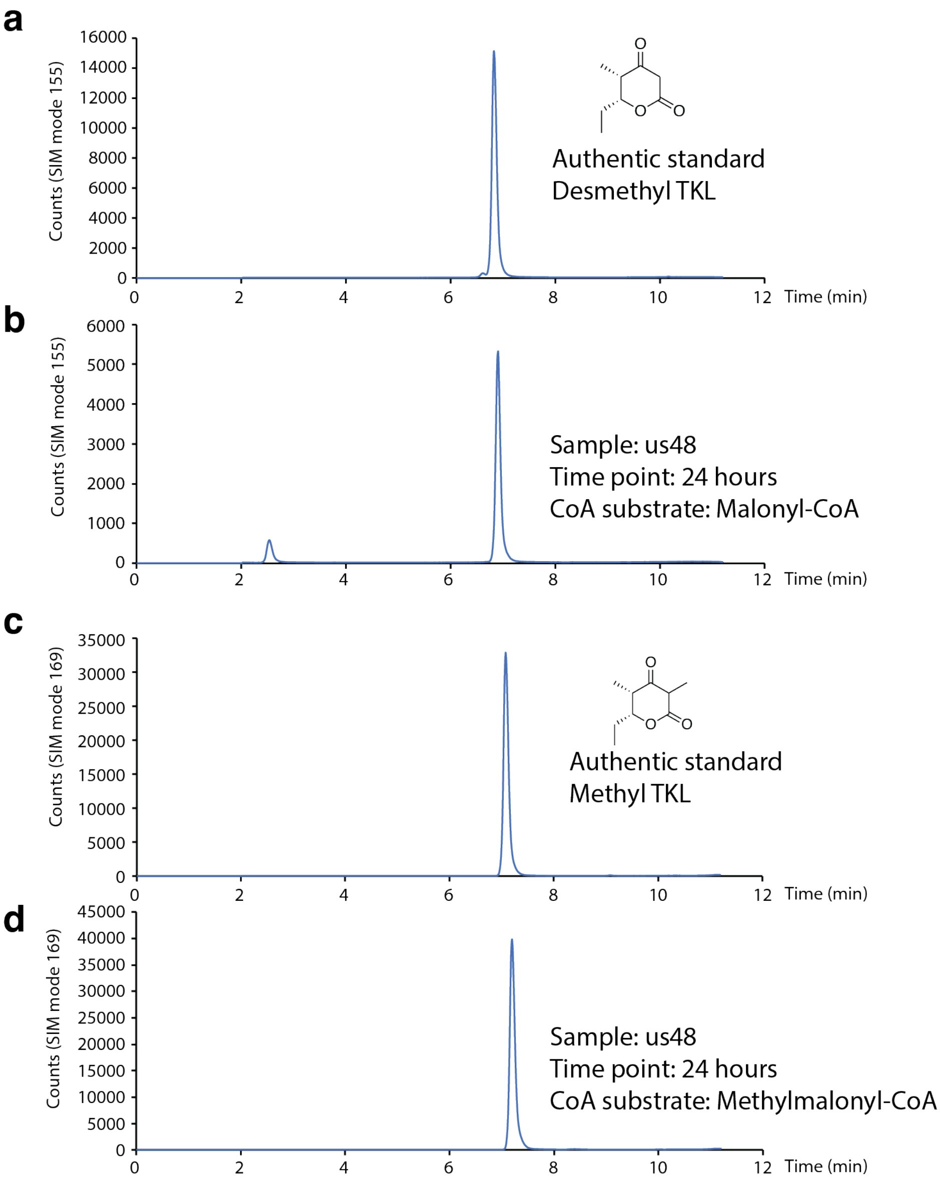

Supplementary Fig. 8: LC-MS analysis of authentic standards of (a) desmethyl and (c) methyl TKL and (b, d) a representative *in vitro* sample producing both products (us48 from Fig 4e, f). Formation of the product were monitored at mass 155 for desmethyl and 169 for methyl TKL. A standard curve was constructed from the standards and areas were compared to quantify *in vitro* production.

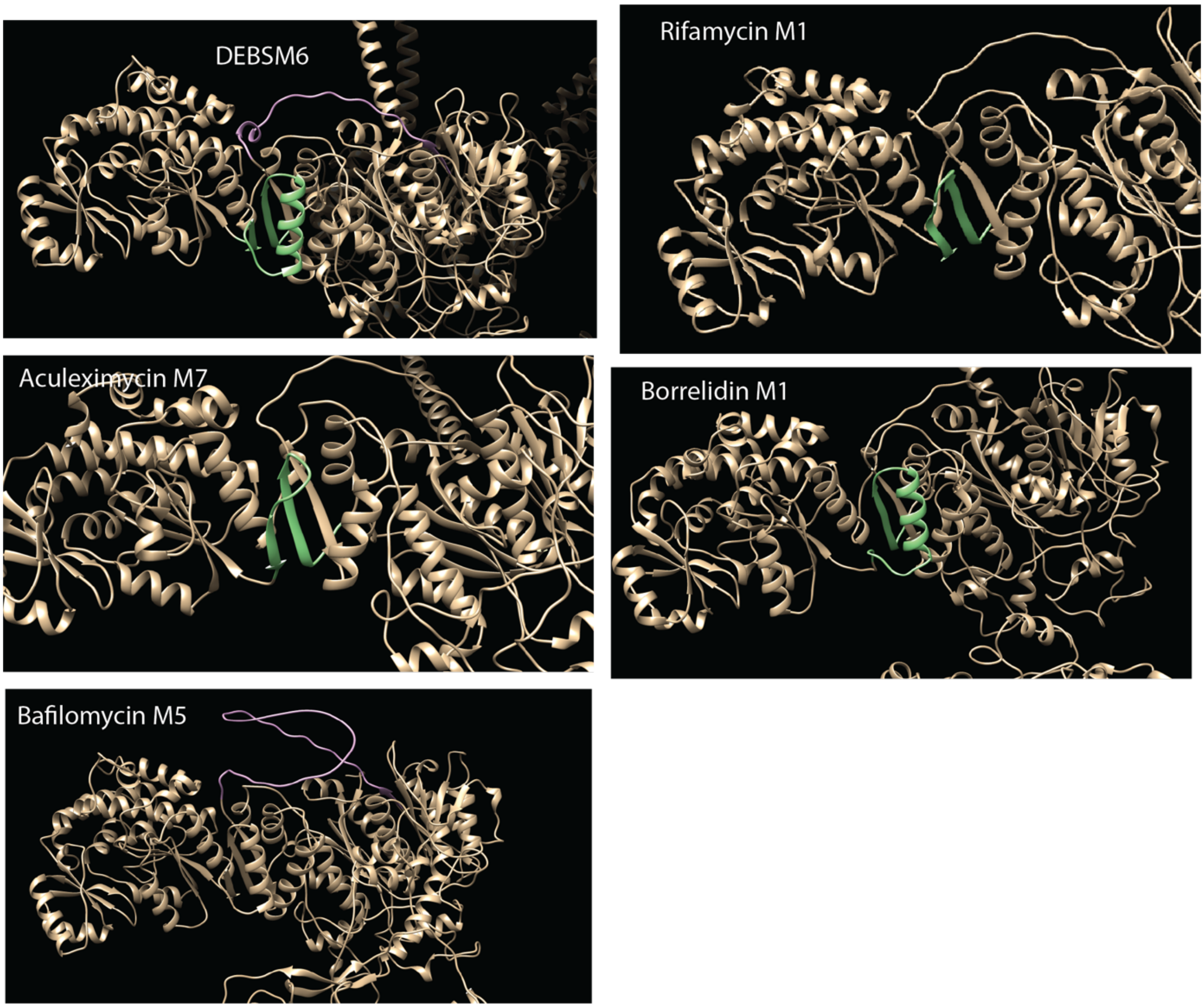

Supplementary Fig. 9. Unusual KS-AT linker structures from AlphaFold models. Structure of DEBSM6 (top left) as reference has α0-β0 residues marked in pink and β2, α3 and β3 marked in green. Rifamycin M1 (top right) and aculeximycin M7 (mid left) lacks α3, borrelidin M1 (mid right) lacks β3 and bafilomycin M5 (bottom left) has a 25 amino acid insertion into α0-β0 linker.

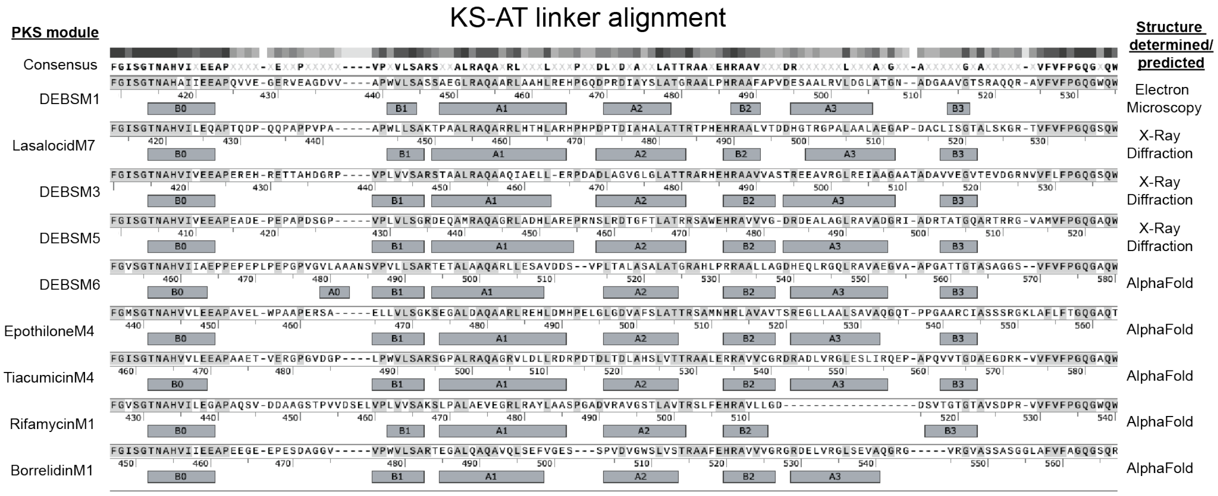

Supplementary Fig. 10. Sequence alignment of KS-AT linker region to verify that secondary structures of experimentally determined models align with AlphaFold predicted secondary structures. SnapGene was used to generate alignment using MUSCLE algorithm. The four experimentally determined models are DEBSM1[2], LasM7[3], DEBSM3[4], DEBSM5[5].

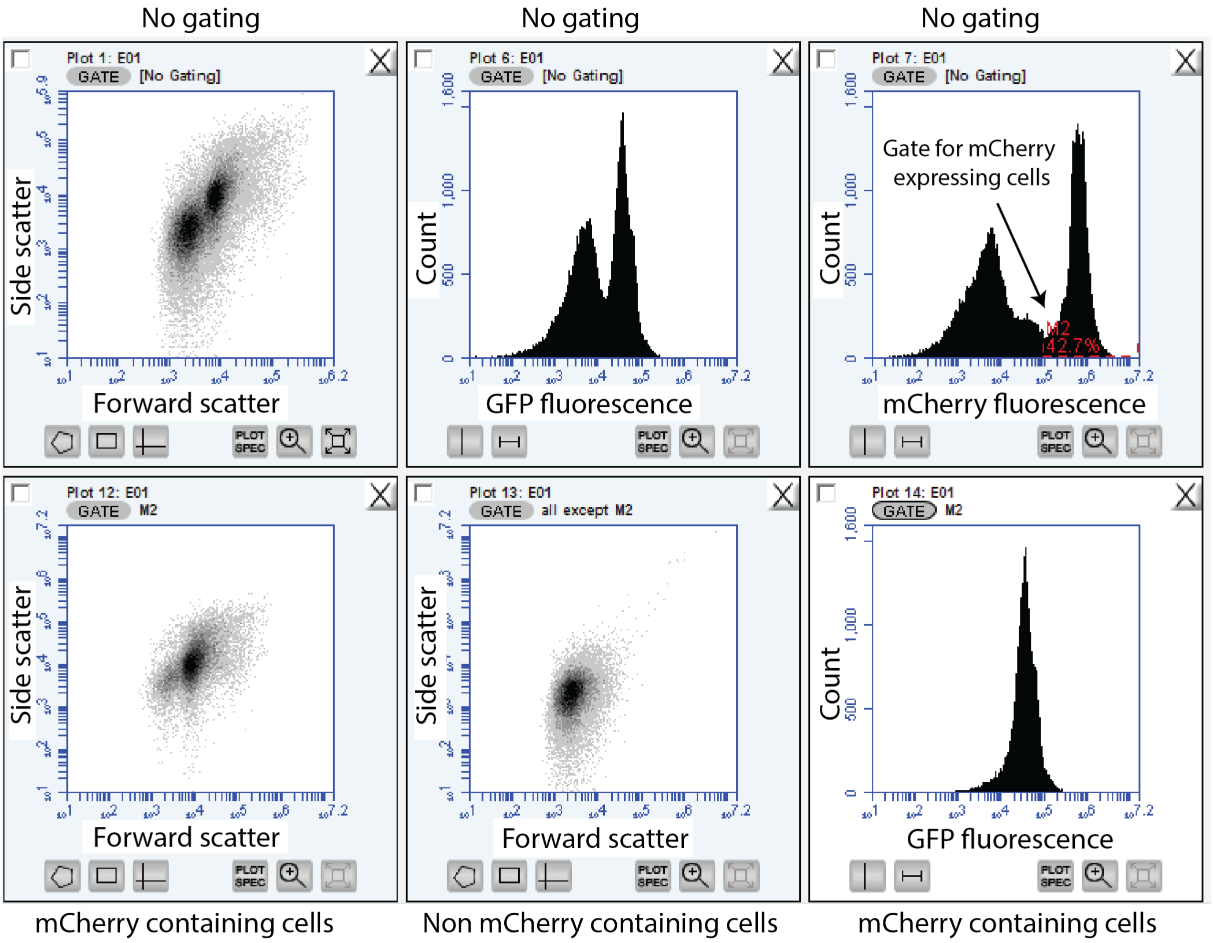

Supplementary Fig. 11. Flow cytometry results showing example of bimodal distribution of cell subpopulations with and without mCherry. Example shows results from D1 mCherry. A gate for cells with higher mCherry fluorescence (top right) was applied to extract GFP fluorescence only from mCherry expressing cells (bottom right).

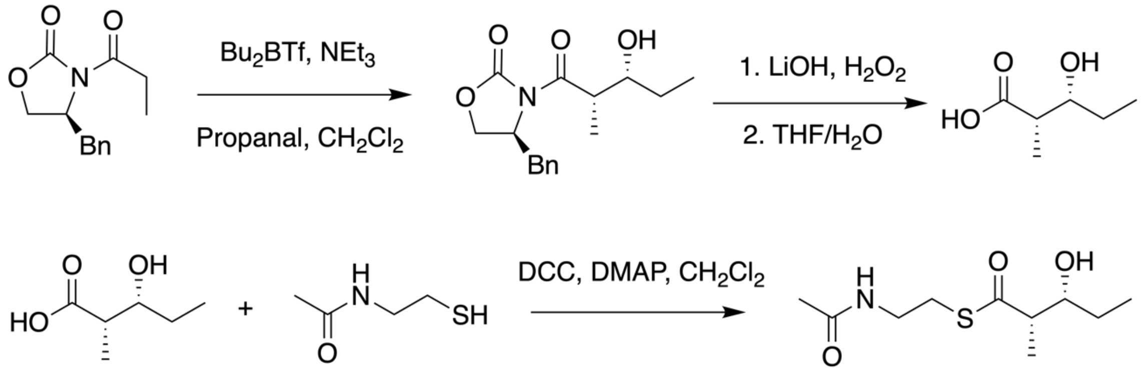

1

Supplementary Fig. 12. Reaction scheme for synthesis of (2S,3R)-3-hydroxy-2-methylpentanoyl-S-N-acetylcysteamine thioester (**1**), based on Yuzawa et al. 2017[6] and Sharma & Boddy 2007[7].

High solubility colony	Upstream Junction	Downstream junction
**o01**	us19	ds24
**o02**	us14	ds61
**o03**	us11	ds28
**o04**	us85	ds21
**o05**	us18	ds62c
**o06**	us33	ds58
**o07**	us16	ds61
**o08**	u13	ds43
**o09**	us18	ds51
**o10**	us18	ds62h
**o11**	us14	ds28
**o12**	us26	ds62h
**o13**	us43	ds46
**o14**	us17	ds65
**o15**	us21	ds64
**o16**	us19	ds51
**o17**	us28	ds62e
**o18**	us43	ds56
**o19**	us25	ds58
**o20**	us10	ds38
**o21**	us11	ds43
**o22**	us36	ds51
**o23**	us19	ds60
**o24**	us39	ds40
**o25**	us82	ds24
**o26**	us86	ds41
**o27**	us32	ds61
**o28**	us14	ds62c
**o29**	us14	ds56
**o30**	us33	ds62e
**o31**	us33	ds57
**o32**	us22	ds41
**o33**	us17	ds25
**o34**	us21	ds62d
**o35**	us18	ds62a
**o36**	us20	ds56
**o37**	us86	ds19
**o38**	us20	ds40
**o39**	us43	ds61
**o40**	us10	ds62e

Supplementary Table 1. Junction position of high solubility colonies.

PKS	Module	Domains in module	Modeled domains	AT substrate
Erythromycin	6	KS-AT-KR-ACP-TE	KS-AT-KR-ACP	Methylmalonyl-CoA
Epothilone	1	KS-AT-KR-ACP	KS-AT-KR	Methylmalonyl-CoA + malonyl-CoA
Tiacumicin	4	KS-AT-KR-ACP	KS-AT-KR	Ethylmalonyl-CoA
Lipomycin	1	KS-AT-KR-ACP	KS-AT-KR	Methylmalonyl-CoA
Borrelidin	1	KS-AT-KR-ACP	KS-AT-KR	Malonyl-CoA
Rifamycin	1	KS-AT-DH-ER-KR-ACP	KS-AT-DH	Methylmalonyl-CoA
Aculeximycin	7	KS-AT-DH-KR-ACP	KS-AT-DH	Malonyl-CoA
Bafilomycin	5	KS-AT-KR-ACP	KS-AT-KR	Methoxy-ACP
Filipin	13	KS-AT-KR-ACP	KS-AT-KR	Hexylmalonyl.CoA
Lasalocid	1	KS-AT-DH-KR-ACP	KS-AT-DH	Ethylmalonyl-CoA
Meilingmycin	3	KS-AT-ACP	KS-AT-ACP	Malonyl-CoA
Nemadectin	2	KS-AT-KR-ACP	KS-AT-KR	Methylmalonyl-CoA
Salinomycin	3	KS-AT-DH-ER-KR-ACP	KS-AT-DH	Malonyl-CoA
Aldgamycin	2	KS-AT-KR-ACP	KS-AT-KR	Methylmalonyl-CoA
Chalcomycin	1	KS-AT-KR-ACP	KS-AT-KR	Methylmalonyl-CoA
Hygrcin	7	KS-AT-ACP	KS-AT-ACP	Methylmalonyl-CoA

Supplementary Table 2. AlphaFold models generated in this study. Pdb files can be found in Supplementary File 3.

Name	Plasmid ID	Strain ID	Source
**DEBSM6-TE**	JPUB_005999	JPUB_005998	Yuzawa et al. 2016[6]
**D0**	JPUB_006011	JPUB_006010	Yuzawa et al. 2016[6]
**D1**	JPUB_006001	JPUB_006000	Yuzawa et al. 2016[6]
**DEBSM6 mCherry**	JPUB_019602	JPUB_019601	This study
**D0 mCherry**	JPUB_019604	JPUB_019603	This study
**D1 mCherry**	JPUB_019606	JPUB_019605	This study
**DEBSM6 swap junction library**	JPUB_019608	JPUB_019607	This study
**DEBS EposAT o04**	JPUB_019610	JPUB_019609	This study
**DEBS EposAT o08**	JPUB_019612	JPUB_019611	This study
**DEBS EposAT o15**	JPUB_019614	JPUB_019613	This study
**DEBS EposAT o17**	JPUB_019616	JPUB_019615	This study
**DEBS EposAT o33**	JPUB_019618	JPUB_019617	This study
**DEBS EposAT us1**	JPUB_019620	JPUB_019619	This study
**DEBS EposAT us3**	JPUB_019622	JPUB_019621	This study
**DEBS EposAT us26**	JPUB_019624	JPUB_019623	This study
**DEBS EposAT us27**	JPUB_019626	JPUB_019625	This study
**DEBS EposAT us48**	JPUB_019628	JPUB_019627	This study
**DEBS EposAT us52**	JPUB_019630	JPUB_019629	This study
**DEBS EposAT us79**	JPUB_019632	JPUB_019631	This study
**DEBS EposAT us84**	JPUB_019634	JPUB_019633	This study
**Δ*arsB* P_{ibp} GFP**	N/A	JPUB_019635	This study
**Δ*arsB* P_{ibpfxs} GFP**	N/A	JPUB_019636	This study
**Δ*ibpA gfp***	N/A	JPUB_019637	This study

Supplementary Table 3. Plasmids and strains used in this study. All sequences can be downloaded, and strains physically requested at: https://public-registry.jbei.org/folders/706

Supplementary References

1 Bachmann, B. O. & Ravel, J. In silico prediction of microbial secondary metabolic pathways from DNA sequence data. *Methods Enzymol.* **458**, 181-217 (2009).

2 Cogan, D. P. *et al.* Mapping the catalytic conformations of an assembly-line polyketide synthase module. *Science* **374**, 729-734 (2021).

3 Bagde, S. R., Mathews, I. I., Fromme, J. C. & Kim, C.-Y. Modular polyketide synthase contains two reaction chambers that operate asynchronously. *Science* **374**, 723-729 (2021).

4 Tang, Y., Chen, A. Y., Kim, C.-Y., Cane, D. E. & Khosla, C. Structural and mechanistic analysis of protein interactions in module 3 of the 6-deoxyerythronolide B synthase. *Chem. Biol.* **14**, 931-943 (2007).

5 Tang, Y., Kim, C.-Y., Mathews, I. I., Cane, D. E. & Khosla, C. The 2.7-Å crystal structure of a 194-kDa homodimeric fragment of the 6-deoxyerythronolide B synthase. *Proc. Natl. Acad. Sci.* **103**, 11124-11129 (2006).

6 Yuzawa, S. *et al.* Comprehensive in vitro analysis of acyltransferase domain exchanges in modular polyketide synthases and its application for short-chain ketone production. *ACS synth. biol.* **6**, 139-147 (2017).

7 Sharma, K. K. & Boddy, C. N. The thioesterase domain from the pimaricin and erythromycin biosynthetic pathways can catalyze hydrolysis of simple thioester substrates. *Bioorganic Med. Chem. Lett.* **17**, 3034-3037 (2007).
