# Supplementary material for: Biosensor Guided Polyketide Synthases Engineering for Optimization of Domain Exchange Boundaries
Source: Nat Commun. 2023 Aug 12;14:4871. doi: 10.1038/s41467-023-40464-x (PMC10423236; doi:10.1038/s41467-023-40464-x)
Supplement: Supplementary file 3 — Description of Additional Supplementary Files Document [file 41467_2023_40464_MOESM3_ESM.pdf]

Description of Additional Supplementary Files

Supplementary Data 1: Oligo pool library sequences
Supplementary Data 2: KS-AT solubility data
