# Supplementary material for: Biosensor Guided Polyketide Synthases Engineering for Optimization of Domain Exchange Boundaries
Source: Nat Commun. 2023 Aug 12;14:4871. doi: 10.1038/s41467-023-40464-x (PMC10423236; doi:10.1038/s41467-023-40464-x)
Supplement: Supplementary file 6 — Reporting Summary [file 41467_2023_40464_MOESM6_ESM.pdf]

nature portfolio

Corresponding author(s): Jay D. Keasling

Last updated by author(s): Jul 24, 2023

Reporting Summary

Nature Portfolio wishes to improve the reproducibility of the work that we publish. This form provides structure for consistency and transparency in reporting. For further information on Nature Portfolio policies, see our Editorial Policies and the Editorial Policy Checklist.

Statistics

For all statistical analyses, confirm that the following items are present in the figure legend, table legend, main text, or Methods section.

n/a	Confirmed	
☐	☒	The exact sample size (*n*) for each experimental group/condition, given as a discrete number and unit of measurement
☐	☒	A statement on whether measurements were taken from distinct samples or whether the same sample was measured repeatedly
☒	☐	The statistical test(s) used AND whether they are one- or two-sided<br>*Only common tests should be described solely by name; describe more complex techniques in the Methods section.*
☒	☐	A description of all covariates tested
☒	☐	A description of any assumptions or corrections, such as tests of normality and adjustment for multiple comparisons
☐	☒	A full description of the statistical parameters including central tendency (e.g. means) or other basic estimates (e.g. regression coefficient) AND variation (e.g. standard deviation) or associated estimates of uncertainty (e.g. confidence intervals)
☒	☐	For null hypothesis testing, the test statistic (e.g. *F*, *t*, *r*) with confidence intervals, effect sizes, degrees of freedom and *P* value noted<br>*Give P values as exact values whenever suitable.*
☒	☐	For Bayesian analysis, information on the choice of priors and Markov chain Monte Carlo settings
☒	☐	For hierarchical and complex designs, identification of the appropriate level for tests and full reporting of outcomes
☒	☐	Estimates of effect sizes (e.g. Cohen's *d*, Pearson's *r*), indicating how they were calculated

Our web collection on statistics for biologists contains articles on many of the points above.

Software and code

Policy information about availability of computer code

Data collection	Protein bands were quantified using VisionWorks (Analytik Jena), flow cytometry data was collected using BD accuri C6 Plus software (BD Bioscience). Alphafold v2.0.1 was used with modifications according to Colabfold. Additional code to run AlphaFold program can be downloaded from https://github.com/Alberto024/alphafold. Sequence alignment was performed using Clustal Omega (https://www.ebi.ac.uk/Tools/msa/clustalo/) or by Snapgene version 7.0.1.
Data analysis	*Provide a description of all commercial, open source and custom code used to analyse the data in this study, specifying the version used OR state that no software was used.*

For manuscripts utilizing custom algorithms or software that are central to the research but not yet described in published literature, software must be made available to editors and reviewers. We strongly encourage code deposition in a community repository (e.g. GitHub). See the Nature Portfolio guidelines for submitting code & software for further information.

Data

Policy information about availability of data

All manuscripts must include a data availability statement. This statement should provide the following information, where applicable:

- Accession codes, unique identifiers, or web links for publicly available datasets
- A description of any restrictions on data availability
- For clinical datasets or third party data, please ensure that the statement adheres to our policy

A list of all plasmids used in this paper can be found in Supplementary Table 3 and plasmid sequences and strain request is available at the Joint BioEnergy Institute's public Inventory of Composable Elements (https://public-registry.jbei.org). Source data is available as a Source Data file for all figures. Source data also contains pdb structures of AlphaFold models, DNA sequences of biosensors and raw data files from a subset of LC-MS samples. The complete data set from Fig. 5b with solubility values and protein sequences are available as Supplementary Data 2.

Research involving human participants, their data, or biological material

Policy information about studies with human participants or human data. See also policy information about sex, gender (identity/presentation), and sexual orientation and race, ethnicity and racism.

Reporting on sex and gender	N/A
Reporting on race, ethnicity, or other socially relevant groupings	N/A
Population characteristics	N/A
Recruitment	N/A
Ethics oversight	N/A

Note that full information on the approval of the study protocol must also be provided in the manuscript.

Field-specific reporting

Please select the one below that is the best fit for your research. If you are not sure, read the appropriate sections before making your selection.

☒ Life sciences ☐ Behavioural & social sciences ☐ Ecological, evolutionary & environmental sciences

For a reference copy of the document with all sections, see nature.com/documents/nr-reporting-summary-flat.pdf

Life sciences study design

All studies must disclose on these points even when the disclosure is negative.

Sample size	All experiments used at least 3 biological replicates as indicated in the figure legends.
Data exclusions	No data was excluded
Replication	Biological triplicates were used for measured data. SDS-PAGE of purified proteins were done only once.
Randomization	Randomization was not performed in our experiments. All samples for each group was sampled under the same conditions.
Blinding	Blinding was not used in our experiments. All samples for each group was sampled under the same conditions.

Reporting for specific materials, systems and methods

We require information from authors about some types of materials, experimental systems and methods used in many studies. Here, indicate whether each material, system or method listed is relevant to your study. If you are not sure if a list item applies to your research, read the appropriate section before selecting a response.

Materials & experimental systems

n/a	Involved in the study
☒	☐ Antibodies
☒	☐ Eukaryotic cell lines
☒	☐ Palaeontology and archaeology
☒	☐ Animals and other organisms
☒	☐ Clinical data
☒	☐ Dual use research of concern
☒	☐ Plants

Methods

n/a	Involved in the study
☒	☐ ChIP-seq
☐	☒ Flow cytometry
☒	☐ MRI-based neuroimaging

Flow Cytometry

Plots

Confirm that:

☒ The axis labels state the marker and fluorochrome used (e.g. CD4-FITC).

☒ The axis scales are clearly visible. Include numbers along axes only for bottom left plot of group (a 'group' is an analysis of identical markers).

☒ All plots are contour plots with outliers or pseudocolor plots.

☒ A numerical value for number of cells or percentage (with statistics) is provided.

Methodology

Sample preparation	E. coli cells were grown with 250 uM IPTG at 250 rpm, 18C for 20 hours. 10 ul of culture was diluted with 150 ul PBS and sampled in the flow cytometer.
Instrument	BD Accuri C6 (BD Bioscience)
Software	BD accuri C6 Plus software (BD Bioscience)
Cell population abundance	50000 events were collected.
Gating strategy	Cells was gated to include only the ones that had over 10^5 mCherry fluorescence. See Supplementary Fig. 8 for details.

☒ Tick this box to confirm that a figure exemplifying the gating strategy is provided in the Supplementary Information.
